# A Proposal for a Patient-Oriented Five-Dimensional Approach for Surveillance and Therapy in Multiple Sclerosis

**DOI:** 10.3389/fneur.2017.00313

**Published:** 2017-07-03

**Authors:** Yavor Yalachkov, Christian Foerch, Mathias Wahl, Johannes Gehrig

**Affiliations:** ^1^Department of Neurology, Goethe-University, Frankfurt am Main, Germany

**Keywords:** multiple sclerosis, disease-modifying therapy, therapy monitoring, patient safety, clinical tool

## Introduction

The former (1) and current (2) multiple sclerosis (MS) classifications are essential for describing different phenotypes and disease dynamics. To establish personalized treatment regimes, further clinical and paraclinical parameters have to be considered such as imaging, cerebrospinal fluid (CSF) findings, past disease-modifying therapies (DMTs), and disease activity under these therapies. In clinical practice, this information is often difficult to overview. Especially, patients with a long course of disease offer an extensive medical history so that comprehending all of the necessary information can be very time consuming.

## The Five-Dimensional Approach for Surveillance and Therapy (FAST) Tool

We suggest the introduction of a FAST into the clinical practice of MS. This tool aims at supporting neurologists in monitoring and treating MS patients. It recognizes the achievements of the former and current MS classifications and includes additional patient- and disease-related information in a five-dimensional structure to inform the clinician and deliver the necessary arguments for therapy selection. FAST has been partially inspired by a five-dimensional patient-oriented classification in epileptic patients (3), which uses medical history as well as clinical and paraclinical information to classify patients into particular epilepsy groups.

### Dimension 1: Phenotype

The first dimension of FAST is defined as “phenotype.” Here, we retain the already well-established MS phenotypes [“relapse-remitting,” “secondary progressive,” “primary progressive,” and “clinically isolated syndrome” (CIS)]. Although a “radiologically isolated syndrome” (RIS) has not been recognized as a distinct MS syndrome (2), we believe that including RIS in the first dimension of FAST would facilitate surveillance and, if necessary, therapy of patients whose incidental imaging findings suggest inflammatory demyelination in the absence of symptoms.

### Dimension 2: Disease Dynamics

“Disease dynamics” represents the second dimension of FAST. Besides the current Expanded Disability Status Scale (EDSS), three well-known modifiers from the present MS classification (2) are considered here. “Activity” is defined by clinical or imaging findings and divides the different disease courses in *not active, active*, and *highly active*. “Progression” refers to relapse-independent clinical progression and can be *present* or *missing*. “Worsening” is used to document an increase in disability resulting either from a relapse or from progression.

### Dimension 3: Diagnostic Information

The third dimension of FAST comprises individual findings, which have led to the diagnosis of CIS or MS. This includes the time point of first symptom occurrence, the time point of first diagnosis, information about the McDonald/MAGNIMS criteria (4, 5), and results from laboratory diagnostics including the CSF analysis. Such information is aimed at supporting the treating neurologist in making therapeutic decisions. For example, DMT is indicated in CIS patients in the presence of factors linked to a higher risk of conversion to definite MS (e.g., spinal T2-lesions, >5 cerebral T2-lesions, or oligoclonal banding in CSF).

### Dimension 4: DMT

The fourth dimension, “DMT,” covers the history of DMTs received by the patient, including the current one. It informs the clinician about the individual dynamics of the disease, both clinically and on imaging modalities, and thus indirectly points to the patient-related efficacy of the respective treatment. Furthermore, it may help to recognize patterns of responsiveness to B- or T-cell directed DMTs. Relevant parameters for surveillance of the current DMT like anti-JC virus antibody serum levels or lymphocyte count can be integrated in this dimension, enabling a more stringent monitoring of therapy-associated risks.

### Dimension 5: Related Medical Conditions

The fifth dimension, “related medical conditions,” describes individual aspects that influence the therapeutic decision or suggest a specific surveillance of the patient. Among others, this includes family planning as well as diseases relevant in the context of certain DMTs like liver cirrhosis, hyperthyroidism, and renal insufficiency. Careful consideration of the information represented in this final dimension would allow the clinician to choose the DMT with the least side effects and risks for this patient.

## Example of Using FAST in the Clinical Practice

To demonstrate the benefits of FAST in the clinical context, we present an excerpt from an extensive medical report on a fictive MS patient who seeks a second opinion about escalation of her DMT. After having read the medical history, the reader may compare it directly with the content of Table 1. It shows how the most relevant information can be summarized in a structured way, providing the clinician both with essential arguments for treatment recommendations and a well-arranged monitoring tool.

**Table 1:** On the left side of the table are the five dimensions of FAST with the available options which the clinician can choose between (e.g., “Phenotype” can be “relapse-remitting,” “secondary progressive,” etc.) and the different subdimensions [e.g., “Disease dynamics” is defined by information about the current Expanded Disability Status Scale (EDSS), activity, progression, and worsening].

**Patient name**: **Age**: 1. **Phenotype** – Relapse-remitting disease – Secondary progressive disease – Primary progressive disease – Clinically isolated syndrome – Radiologically isolated syndrome 2. **Disease dynamics** 2.1 Current EDSS 2.2 Activity (clinical/imaging) – Not active – Active – Highly active 2.3 Progression (clinical) – Without progression – With progression 2.4 Worsening (clinical) – Worsening – Stable 3. **Diagnostic information** 3.1 Time of first symptoms 3.2 Time of first diagnosis 3.3 Revised McDonald criteria 3.4 Cerebrospinal fluid (CSF) and other relevant laboratory findings 4. **Disease-modifying therapy (DMT)** 4.1 Current DMT (xxxx–until now) – Annualized relapse rate or progression during this period – Other relevant aspects (e.g., anti-JC-virus antibody serum levels) 4.2 Previous DMT 1 (xxxx–xxxx) – Annualized relapse rate or progression during this period – Other relevant aspects (e.g., anti-JC-virus antibody serum levels) 4.2 Previous DMT 2 (xxxx–xxxx) 5. **Related medical conditions**	**Patient name**: X **Age**: 27 1. **Phenotype**: relapse-remitting disease 2. **Disease dynamics** 2.1 *Current EDSS*: 3 2.2 *Activity*: active [two relapses 2016–2017 (one optic neuritis and one transverse myelitis) as well as a new MRI lesion] 2.3 *Progression*: without progression 2.4 *Worsening*: worsening (2016–2017: EDSS increase from 2 to 3 due to growing vision loss of the left eye) 3. **Diagnostic information** 3.1 *Time of first symptoms*: 01/2010 3.2 *Time of first diagnosis*: 05/2012 3.3 *Revised McDonald criteria*: – Clinical criteria for dissemination in time and space are met – MAGNIMS criteria are met 3.4 *CSF and other relevant laboratory findings*: – *05/2012*: 17 leukocytes/μl, oligoclonal banding – *06/2016*: 14 leukocytes/μl, oligoclonal banding, JCV-PCR negative, aquaporin-4-antibodies negative in serum and CSF 4. **DMT** 4.1 Current DMT (01/2016–until now): dimethyl fumarate – Annualized relapse rate: 2/year – Other relevant aspects: Lymphocyte count 01/2017: 1.69/nl Anti-JCV antibody serological index 01/2016: negative 01/2017: negative 4.2 Previous DMT (05/2012–12/2016): glatiramer acetate – Annualized relapse rate: 2/year – Other relevant aspects: mild skin irritation reaction 5. **Related medical conditions** – Hashimoto’s thyroiditis – Patient is planning to have a child in the next 1 or 2 years.

*An example for a fictional patient and how the five-dimensional approach for surveillance and therapy (FAST) can be applied in the clinical practice is shown on the right side of the table*.

### Excerpt from a Medical Report

This patient is a 27-year-old woman suffering from a relapsing–remitting MS with a current EDSS score of 3. First symptoms occurred in 2010, and she was diagnosed with MS in May 2012 according to the revised McDonald criteria (5). Disease activity is quantified as two relapses during the last year (an optic myelitis and a transverse myelitis) and a new contrast-enhancing T1 hyperintense lesion. There is currently no evidence for a relapse-independent progression, but she accumulated disability due to residual deficits resulting from her last relapses (her EDSS increased from 2 to 3 after a remarkable vision loss of the left eye due to a therapy-refractory optic neuritis). Dissemination of inflammatory activity both in time and space was clinically detected. The MAGNIMS imaging criteria were also met (4). Alternative diagnoses were ruled out. CSF was analyzed in May 2012 and in June 2016. Results were compatible with the diagnosis. The second CSF analysis included a polymerase chain reaction for JCV to rule out a progressive multifocal leukoencephalopathy (PML), since the patient exhibited an atypical lesion in one of the follow-up brain MRIs. Due to the occurrence of both optic neuritis and transverse myelitis, aquaporin-4 antibodies were analyzed in CSF and serum (negative). Thus, both neuromyelitis optica (NMO) and NMO-spectrum disorder appeared unlikely (MAGNIMS criteria were met, T2-hyperintense lesions were typical for MS, and there was no indication for a longitudinal myelitis). Her first DMT was glatiramer acetate that she took for about 4 years without severe side effects except for a mild skin irritation. Relapse rate during therapy was 2/year. Since January 2016 the DMT was switched to dimethyl fumarate but her relapse rate remained the same. Lymphocyte count was regularly measured, and the last results were within the normal range. Due to the still unchanged relapse rate and the accumulation of disability, escalation DMT has been suggested.

### Feeding the Information from the Report into FAST

Besides the essential information extracted from the above presented medical history, the neurologist may feed further patient-related information into FAST, which is relevant to facilitate DMT decision as well as to monitor the further therapy. For example, any plans of the patient to have children in the near future may be documented so that therapies with presumed teratogenicity may be avoided or timely discontinued. Disease duration (relevant in patients with disease duration <10 years) can also be fed into FAST and further updated. Any relevant concomitant medical condition that might reduce the individual risk–benefit ratio from a particular DMT (such as Hashimoto’s thyroiditis and liver disease) can also be documented. Anti-JCV antibody serological index that increases the risk for developing a PML under escalation DMTs can be documented and monitored using FAST.

Table 1 shows FAST in its default format and how it looks like when the information from the medical report is fed into the tool itself. Obviously, the information in FAST can be effortlessly updated when new aspects arise (e.g., relapses, medical results, etc.). This stresses the advantage of using FAST in clinical practice. It does not only serve as a tool to keep the relevant patient-related information continuously updated but may also facilitate communication when patients are referred by neurologists to MS treatment centers for a second opinion.

## Discussion

Five-dimensional approach for surveillance and therapy does not replace the current MS classification, the EDSS, the neurologist’s medical report, or MS registries and databases. Strictly speaking, it does not offer new elements beyond the knowledge that neurologists already apply in their daily practice. It rather offers an opportunity for the treating specialist to improve the organization of clinical reports, while having all relevant information in one place, easily accessible for a quick overview, thus facilitating surveillance and therapeutic decision making. Well-designed registries and databases offer more detailed information on the disease course and are an essential prerequisite for scientific analyses. However, they are less practicable when it comes to the extraction and weighting of individual data in daily routine.

Introducing FAST in clinical practice offers four main advantages. First, it contributes decisively to patient safety by presenting essential therapy-related information in a structured way, which enables easier update of information. Consequently, the treating neurologist is always aware of the individual risks of his/her patients when considering a particular DMT. Thus, monitoring the current therapy is more easily ensured. A widespread use of FAST would simplify information transfer after switching to a new clinic or a new neurologist. Second, using FAST saves time. While extensive medical reports are not replaced by FAST, new patient-related information such as imaging findings, relapses, etc. can be easily fed in the respective dimensions of FAST. Thus, the most relevant patient-associated information is summarized in a condensed and consistent way, allowing the neurologist to access it without having to read the entire medical reports anew. Third, using FAST can be beneficial especially for didactic reasons: neurologists in training can more easily overview the complex information that has to be considered when recommending or monitoring a DMT. This applies also for specialists working in regions with low MS prevalence or in clinics treating only a low number of MS patients. Last but not least, FAST offers a simple and effective way of screening patients with regard to study inclusion criteria. All of the relevant information like phenotype, EDSS, activity, diagnostic criteria, former DMTs, disease duration, and other medical conditions are stored in one tool, where the information is not only presented in a structured and patient-related way but can also be easily updated.

## Author Contributions

YY, CF, MW, and JG developed and discussed the main idea of the manuscript. YY, CF, and JG wrote the manuscript.

## Conflict of Interest Statement

YY and JG have no conflicts of interest. CF received speaking honoraria and honoraria for active participation in advisory boards from TEVA, Roche, and Genzyme. MW’s work has been supported by a research grant from TEVA.

## References

[1] Lublin FD Reingold SC. Defining the clinical course of multiple sclerosis: results of an international survey. National Multiple Sclerosis Society (USA) advisory committee on clinical trials of new agents in multiple sclerosis. Neurology (1996) 46:907–11. 10.1212/WNL.46.4.907 8780061

[2] Lublin FD Reingold SC Cohen JA Cutter GR Sorensen PS Thompson AJ Defining the clinical course of multiple sclerosis: the 2013 revisions. Neurology (2014) 83:278–86. 10.1212/WNL.0000000000000560 24871874 PMC4117366

[3] Loddenkemper T Kellinghaus C Wyllie E Najm IM Gupta A Rosenow F A proposal for a five-dimensional patient-oriented epilepsy classification. Epileptic Disord (2005) 7:308–16. 16338673

[4] Filippi M Rocca MA Ciccarelli O De Stefano N Evangelou N Kappos L MRI criteria for the diagnosis of multiple sclerosis: MAGNIMS consensus guidelines. Lancet Neurol (2016) 15:292–303. 10.1016/S1474-4422(15)00393-2 26822746 PMC4760851

[5] Polman CH Reingold SC Banwell B Clanet M Cohen JA Filippi M Diagnostic criteria for multiple sclerosis: 2010 revisions to the McDonald criteria. Ann Neurol (2011) 69:292–302. 10.1002/ana.22366 21387374 PMC3084507

